# Application of Composite Hydrogels to Control Physical Properties in Tissue Engineering and Regenerative Medicine

**DOI:** 10.3390/gels4020051

**Published:** 2018-05-30

**Authors:** Cassidy Sheffield, Kaylee Meyers, Emil Johnson, Rupak M. Rajachar

**Affiliations:** Department of Biomedical Engineering, Michigan Technological University, Houghton, MI 49931, USA; cmhyde@mtu.edu (C.S.); kmmeyers@mtu.edu (K.M.); emilj@mtu.edu (E.J.)

**Keywords:** composites, hydrogels, controlled physical properties, tissue engineering

## Abstract

The development of biomaterials for the restoration of the normal tissue structure–function relationship in pathological conditions as well as acute and chronic injury is an area of intense investigation. More recently, the use of tailored or composite hydrogels for tissue engineering and regenerative medicine has sought to bridge the gap between natural tissues and applied biomaterials more clearly. By applying traditional concepts in engineering composites, these hydrogels represent hierarchical structured materials that translate more closely the key guiding principles required for improved recovery of tissue architecture and functional behavior, including physical, mass transport, and biological properties. For tissue-engineering scaffolds in general, and more specifically in composite hydrogel materials, each of these properties provide unique qualities that are essential for proper augmentation and repair following disease and injury. The broad focus of this review is on physical properties in particular, static and dynamic mechanical properties provided by composite hydrogel materials and their link to native tissue architecture and, ultimately, tissue-specific applications for composite hydrogels.

## 1. Introduction

The restoration of the biological tissue structure–function relationship is the primary challenge faced in the rational development of regenerative biomaterials and more specifically composite hydrogels scaffolds. It requires the ability of the material to both mimic tissue structure as well as biological, mass transport, and physical properties including static and dynamic mechanical behavior [1,2,3,4]. The later attribute is one of the key limitations of monolith, single-phase hydrogels. Taking into account the hierarchical composite structure–function relationships of native tissues, the design of biomimetic composite hydrogels able to reproduce the multiscale architectural nature of complex tissues represents a crucial unmet need for tissue repair and regeneration.

Biological tissues exhibit a large spectrum of mechanical properties [5]. Monolithic (bio)materials are unable to accommodate this range, making them insufficient in guiding proper repair in injury. The ability to customize the properties to the desired application is where it becomes apparent that composites are a better choice. In traditional engineering materials, improved strength, stiffness, fatigue life, impact resistance, and corrosion resistance can be realized through the use of composites [6]. The same has been shown to be true in the development of composite hydrogels, where properties are being customized to adequately meet specific regenerative and repair requirements in medicine [7,8,9,10,11]. The focus of this review is to provide an overview of composite hydrogels, the rationale of their application, and tissue-specific examples showing how their unique properties may be used to address problems in tissue engineering and regenerative medicine.

## 2. Natural Composites

**Figure 1:**
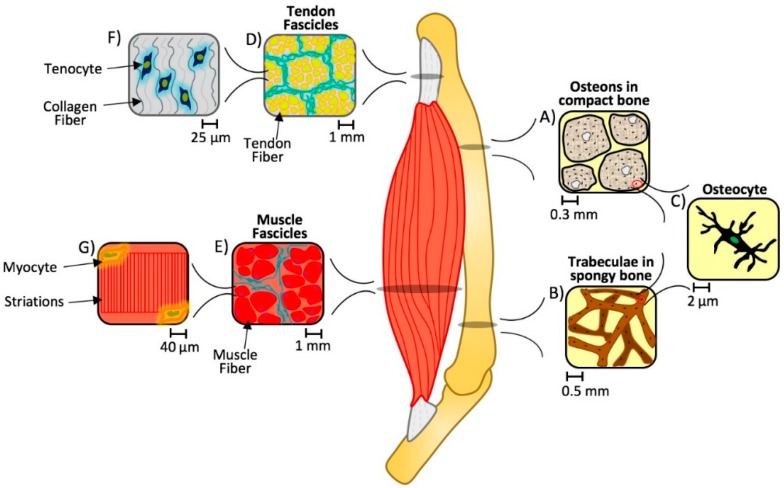
Multiscale hierarchical structure of bone, tendon, and muscle. An organ system consists of several organs with underlying hierarchical structures. In this case, bone (**A**–**C**), tendon (**D**,**F**), and muscle (**E**,**G**) units consist of unique cellular components (in the order of 2–40 µm) that are capable of sensing and modulating the tissue-level composite architecture at various length scales (from 0.3–1 mm, depending on the tissue) under physiological conditions. When these architectures are disturbed, the normal spatial and temporal responses of these tissues to their static and dynamic biomaterial environment is disrupted and contributes to eccentric loading and ultimately injury and pathological losses in structure and function [13,14,15,16,17,18].

Since composite hydrogels are formed from mixtures of distinct constituents with different chemical, physical, and biological properties, they may be regarded as composite materials similarly to how human tissues act as natural composites due to their varying compositions. Organs function as needed depending on the location throughout the body, but taking a closer look, the functionality of these organs stem from the natural hierarchy of elemental components i.e., osteons in bone, fascicles in ligament, tendon, and muscle. Each structural unit of these natural biomaterials can range in stiffness, elasticity, density and component orientation, allowing the overarching organ to serve a unique purpose. Figure 1 illustrates how model tissues such as bone, muscle, and connective tissue exhibit distinct composite structures at a macro and micro scale, from the tissue to the cellular level.

Bones are made up of cells in a mineralized organic matrix which enables the tissue to support normal functions such as providing a site for hemopoiesis or mineral storage. Yet this base composite material takes on different spatial configurations within the same bone at the tissue level. Mineral and collagen-based matrices form parallel or twisted lamellar structures that are further organized at the tissue and organ level into compact and spongy bone. While compact bone (Figure 1A) forms a protective shell around the exterior, spongy bone (Figure 1B) is found towards the interior medullary cavity and within the condyles of long bones. At the cellular level, they are similar, made up of a common repeating unit, consisting of osteocytes connected via an extensive canalicular network (Figure 1C). However, compact bone is macroscopically dense, the osteocytes are organized into osteons, or a cylindrical lamellar structure. Whereas spongy bone is a relatively light, macroscopically porous bone, with a disorganized three-dimensional lattice-like trabecular structure. These types of bone have unique attributes to aid in maintaining biomechanical homeostasis in a dynamic mechanical environment [12].

Tendon and muscle also exhibit a hierarchical structure. When looking at a cross section of each, the fibers are arranged into circular bundles, or fascicles (Figure 1D,E). However, on the cellular level, tenocytes are found in a matrix of crimped collagen fibers (Figure 1F), whereas myocytes are located along the edges of the linear, striated, individual muscle fibers (Figure 1G). Again, as in bone, each of these tissues has a unique composition and architecture that contributes to the normal physiological function of these tissues. When the properties of these underlying matrices are compromised, i.e., static and dynamic biomaterial properties, we can see loss of higher order function. Recent studies, for example, suggest changes in fascicle matrix stiffness may promote the loss of normal myocyte contractility seen in cerebral palsy, where myocytes are otherwise normal in structure and function but lose their ability for normal contraction due to changes in their local mechanical environment [19,20].

By increasing the confluency between natural tissues and synthetic hydrogels with similar properties, composite materials could provide innovative mechanisms for tissue engineering. Ultimately the successful regeneration and integration of these micro- and macro-structural elements will dictate the level of balanced recovery between normal tissue structure and functional behavior. Targeting this recovery process using composite materials has become an intense area of study, applying traditional concepts in engineering composites toward developing novel injectable and prefabricated hydrogel composites as tissue-engineering and drug-delivery vehicles.

## 3. Synthetic Composites and Their Classification

The development of composite hydrogel materials for biomedical applications parallels the principles of traditional engineering composites. These materials are produced by mixing constituents to form different phases, predominantly consisting of synthetic polymers that may or may not be mixed with natural or modified natural components (e.g., electrospun protein nanofibers, inverse emulsion formed protein nano- and micro-particles). In general, composites include a matrix, the constituent found in greater quantity with properties that are improved upon by one or more reinforcing or secondary phases that are typically stiffer and stronger than the matrix phase [21]. 

Individual reinforcement phases are classified as having either a particulate or fibrous morphology. Composites may also be altered or hybridized to contain both particle and fiber shapes in their reinforcing phases to elicit the advantageous characteristics of both components in a single composite material. Secondary phase(s) of composites are also often modified spatially to create more robust or stable materials depending on which qualities are desired. By overlapping fibers to form a woven, bidirectional arrangement, or by incorporating particles on the surface of fibers, network secondary phases containing composite-within-composite microstructures are established. In addition, variations of the scaling of these reinforcing phases on both a nano and micro level affects the distinct characteristics exhibited by composites.

**Figure 2:**
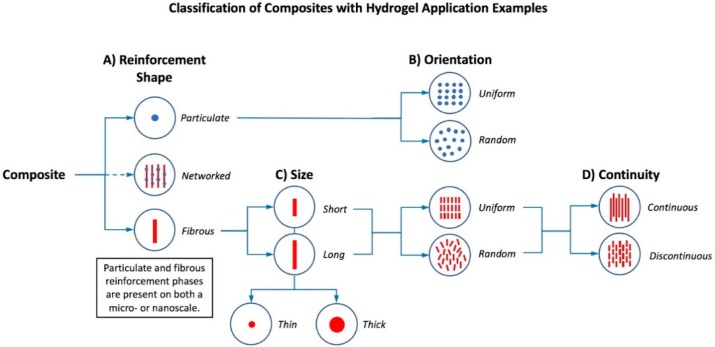
Hierarchical classification of composite hydrogels based on their reinforcing phases at micro and nanoscales. (**A**) Reinforcement shape may be particulate, fibrous, or a hybridization of the two [24,25,26,27,28]; (**B**) both particulate and fibrous reinforcement has either uniform or random orientations [29]; (**C**) fibers in a reinforcing phase can be classified as long or short and further organized based upon fiber diameters or thicknesses; (**D**) within a composite, fibers may have continuous or discontinuous patterns [8].

Specifically, the mechanical properties of composite hydrogels are significantly influenced by the shape, orientation, size, continuity, and composition ratios of their reinforcing phase(s). The shapes of the reinforcing elements generally represent particles or fibers and the orientation of these phases may be random or uniform (Figure 2A,B). Although uniformity in industrial materials is highly desired, composites in a biological setting do not require complete continuity due to the inherent ability of most cell types to remodel their matrix microenvironment as part of normal tissue maintenance (turnover) or in response to injury. Sizes of fibers used in secondary phases may be short or long while fiber diameter could differ as well, both being contingent upon the specific application of the composite (e.g., fibers could provide structural support and a means for load transmission, as well as directional or guiding support for reestablishing anisotropic or directional tissue properties) (Figure 2C). Variations in secondary phase continuity (i.e., continuous or discontinuous patterns) can also be used to influence the strength of the reinforcing phase(s) and patterns of load transmission (Figure 2D). Different matrix-to-reinforcement ratios also alter the features of composite materials due to the unique properties of spatially distinct phases that can be generated (discrete regions of higher and lower concentration of secondary phase elements). These proportions of matrix-to-reinforcement, expressed via weight or volume fractions, enable the manipulation of architectural and mechanical characteristics of a composite material. 

That being said, the region most critical to composite integrity or stability is the matrix-reinforcement interface. Interfacial bonding enables mechanical loads to be transferred from the matrix to the reinforcing phase(s) and vice versa within a composite [21]. In the development of composite hydrogels these interfacial relationships have the same importance. Furthermore, placed in a biological context, these materials and their interactions with both tissues and cells rely on an adhesive interface to the surrounding tissues to promote proper load transfer, and the capacity of cells to detect and transmit forces (i.e., tension, shear, etc.) projected throughout the multiscale hierarchy or organization of a tissue. This maintains normal physiological structure and function within a dynamic mechanical environment via coordinated mechanotransduction and gene expression [12].

In their role for biomedical applications, fibrous and particulate reinforcement phases have distinct physical properties that may provide advantageous enhancements in a composite biomaterial. For example, fibers could supply directionality for cells during regeneration in cardiac or neural tissues, where forms of cellular orientation and organization are essential. In addition, fibrous secondary phases could improve composite mechanical integrity or toughness, yielding a higher strength-to-weight ratio for the material via increased surface area and interfacial bonding. Due to their hierarchical anisotropic nature, fibers may also have unique additional functions such as electrical or thermal conductivity [22]. Conversely, particulate secondary phases impart distinct physical properties including the ability of particles to act as sites for cellular attachment on a micro scale. Factors such as surface morphology and topography of particles largely influence the wetting behavior, extent of protein adsorption, and overall adhesive properties of a composite biomaterial. The chemical and physical structure of particles may also contain hydrophilic-hydrophobic regions or specific biomolecule-binding domains that enable particulate secondary phase(s) to perform as drug-delivery vehicles [23]. Despite their morphological differences, fibrous and particulate reinforcing phases both have the potential to be modified to affect other key physical parameters such as degradation or gelation time. Tuneable physical properties using the intrinsic character of secondary phases lend to the potential effectiveness of composite hydrogels and prospective implications for tissue-engineering and regenerative medicine applications.

## 4. Physical Properties (Static and Dynamic)

### 4.1. Cell Adhesion, Static and Dynamic Physical Environment in Tissues

Factors that have been shown to have a major influence on cell behavior in native and subsequently engineered tissues are: physical, mass transport, and biological properties. For tissue-engineering scaffolds in general, and more specifically in composite hydrogel materials, each of these properties provide unique qualities that are essential for proper augmentation and repair of injury. The narrow focus of this review is on physical properties in particular, and the static and dynamic mechanical properties provided by composite hydrogels materials.

**Figure 3:**
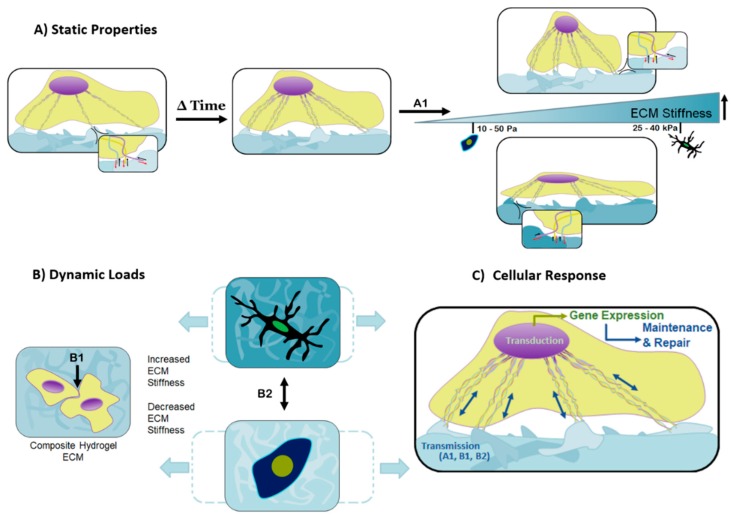
Cellular behavior is affected by a multitude of studied contributions, including mechanical cues, static properties, and dynamic loads. (**A**) Responses to the substrates mechanically static properties are controllable by the matrix stiffness and topographical features, equally capable of dictating cell motility, proliferation, and differentiation. Over time, the cell assesses its local environment and improves focal adhesion sites on suitable substrates through the development of its cytoskeleton. The overall stiffness of the matrix determines the coupled forces created by the probing cell and matrix response, represented by A1. The softer the matrix, the more the cell is able to overcome the matrix stiffness and become rounder as the cytoskeleton pulls inward. In contrast, a matrix of increased stiffness causes the cell to spread over the matrix as the cell’s cytoskeleton is unable to pull inward; (**B**) dynamic forces must appropriately be transferred through the composite substrate for healthy cellular function and ultimately normal tissue- and organ-level turnover. The amplitude and frequency of the dynamic loads, paired with the stiffness of the matrix, determine the maximum deformation of the matrix, represented by B2; (**C**) combining the signals the cell detects from the matrix stiffness, A1, the external dynamic forces, B2, and intercellular contact forces, B1, results in a coordinated transmission, and subsequent transduction (gene expression) that leads to tissue maintenance and repair. These tailorable components are responsible for cellular behavior and can be used in the design of composite hydrogels to create material systems targeted toward diverse cell types and tissues.

The cell-adhesive character and physical properties (e.g., substrate stiffness) of the extracellular microenvironment have been recognized as interdependent factors that influence cell structure and function, as well as normal tissue-level architecture and processes that lead to its maintenance and repair (Figure 3). Consequently, both of these characteristics must be considered when designing hydrogels for tissue-engineering applications. 

The cell internal structure, its cytoskeleton, is connected to the extracellular matrix (ECM) through transmembrane proteins, integrins, that act to anchor or adhere cells to their local environment. Integrins or integrin “receptors” recognize arginine-glycine-aspartic acid (RGD) peptide motifs on proteins in the ECM (e.g., fibronectin and vitronectin) to form strong specific bonds [30,31]. These matrix-adhesion contacts are able to transmit mechanical signals from the ECM to the cell cytoskeleton. For this reason, integrins are often considered to be “cellular mechanosensors” [32,33]. Mechanical loads that are transmitted into the cell are often met with a chemical response; this process is called mechanotransduction, as cells convert mechanical stimuli to a chemical signal (gene expression). Many signaling pathways, such as the mitogen-activated protein kinase/extracellular signal-regulated kinase (MAPK/ERK), Hippo, rho-associated protein kinase (ROCK), and TGFβ-signaling pathways, have been linked to mechanotransduction [34,35,36]. 

Using this interface, cells are not only able to sense but respond to tissue-level stiffness changes as well as transient and cyclical changes in the dynamic mechanical environment. This process allows cells to affect changes that allow them to either maintain or alter their microenvironment to best respond to these changes. For example, differences in local substrate rigidity prime mesenchymal stem cells (MSCs) to move down lineage pathways, i.e., softer matrices coordinated with laminin adhesion can favor neuronal phenotypes whereas stiffer gels promote both neurons as well as astrocytes under similar adhesive conditions. Stiffer matrices have also been shown to promote fibroblastic and osteogenic phenotypic behavior over adipogenic behavior favored in more compliant matrices. These static environmental conditions also affect the local dynamic response of these matrices and, in turn, also influence the tissue-level microenvironment, and ultimately the stability of tissue-level structure and function both in normal and diseased tissues. The exact mechanisms through which cells interpret complex static and dynamic mechanical signals have yet to be elucidated, even though recent discoveries of sensing elements and effectors of ECM mechanical cues such as yes-associated protein/transcriptional coactivator with PDZ-binding motif (YAP/TAZ) have begun to clarify the process that leads from physical forces into biochemical signaling [37,38]. It is however, readily apparent that properly chosen composite hydrogels can act as regenerative guides if their biochemical and physical attributes are tailored to provide an appropriate environment for tissue specific cell adhesion, migration, growth, and differentiation [39,40].

### 4.2. Composite Hydrogels and Role of Static and Dynamic Physical Cues

Composite hydrogels have the potential to increase the success of tissue engineering due to their ability to be easily tailored for a specific function [4,41]. Similar to native cell matrices found throughout the body, the variance in hydrogel structure is accompanied by a corresponding capacity to guide function. This can easily be seen in the tissue-specific response of cells previously mentioned. Bulk properties are recognized by cells on a macro and micro scale and are capable of determining fate, growth, and differentiation [42]. The means by which cells interact with these composite physical properties are dictated, as in natural tissues, by static mechanical properties and dynamic mechanical loading. For example, in nerve regeneration the tendency to grow in linear aligned softer ECM can be compared to fibroblasts which tend to navigate to stiffer, more highly crosslinked ECM [43]. Ultimately, these factors are physical properties targeted in the design of composite hydrogels. 

Some static properties known to contribute to the effectiveness of applied composite hydrogels in wound healing and repair include the surface topography, reinforcement character, and overall structural stiffness [44]. Matrix topography is typically the first encounter cells have with a composite hydrogel. Topographical scapes can range anywhere from grooves and ridges to gaps and holes and, depending on the manufacturing process, are capable of achieving nano to micro scale features [43]. Through careful construction, hydrogels with tuned variance in roughness as well as spatially distinct isotropic and anisotropic features can be achieved [43]. These features have been shown to promote distinct interactions with unique cell types. In addition to the initial encounter between the cell and the matrix surface features, the stiffness of the matrix has also been shown to impact controlled cellular interaction [45].

As a cell navigates through a two or even three-dimensional substrate, the focal tensile forces produced by the cell are coupled with reaction forces from the substrate (Figure 3A). The reaction forces experienced by the cell provide another signaling method which promotes a cascade of internal cellular mechanisms, mainly affecting differentiation and cell type-specific gene expression [43]. Figure 3B illustrates how slight differences in substrate stiffness can dramatically affect cell differentiation, and the coordinated actions of mechano-transmission and mechano-transduction (Figure 3C).

## 5. Biomedical Applications of Composite Hydrogels

There are three fundamental approaches to generating composite hydrogel scaffolds, including: bioprinting, cell encapsulation, and injectable gels [46]. A given techniques must produce a composite that is biocompatible (chemical composition), able to withstand prolonged application specific mechanical forces (macrostructure-microstructure), allows for directed cellular attachment (cell adhesivity), migration (porosity), as well as achieves a conformational architecture with the site of repair [46]. Each key element is necessary to guide the repair and integration of new tissue. Furthermore, these attributes encompass the primary advantages of composite hydrogels in comparison to more traditional hydrogels. These materials can often match compositional biocompatibility, achieve modest directional mechanical properties, and conformational architecture, but quite often lack specificity to actively direct stable tissue repair and regeneration. Composite hydrogels are being investigated in numerous areas of the body: cardiovascular, bone, connective, and neural tissue to name a few. Each application involves the unique choice of component materials, all of which address, in part, the common motifs described above with the goal being to match both structural and functional properties of the desired tissue.

### 5.1. Cardiovascular Applications

Repairing the heart after myocardial infarction is difficult as scar tissue electrically isolates cardiomyocytes leading to dysfunction. The application of hydrogels to repair these tissues looks to reduce the amount of scar tissue as well as to re-establish electrical conductivity. Thus, composite hydrogel materials that can take up some of the load on the tissue during contraction can aid in maintaining tissue volume, reducing scar formation, and promoting healing that preserves cardiac contractility [47]. These materials can also be used as delivery vehicles for cells by providing a stable mechanical environmental for these cells to re-establish functional myocardium [48]. Additionally, several conductive hydrogels have been studied to restore synchronous contraction of the entire heart. Mihic et al. created an injectable polypyrrole chitosan hydrogel [49]. Others are utilizing carbon nanotubes in a collagen or gelatin hydrogel [50,51]. The nanotubes create a channeled highly porous microstructure similar to native heart matrix, and have shown promising in vivo results, enhancing cell alignment and contractility as compared to a non-conductive composite hydrogel. 

Repairing heart valves comes with a whole new set of challenges. The mechanical and bioprosthetic heart valves used today have limitations which include the need for anticoagulation treatments, poor durability, calcification, immunogenic complications, and the lack of remodeling and ability to grow with the patient [52]. Previous approaches used isotropic homogeneous scaffolds which do not match the anisotropic properties and laminate structure of valves. Tseng et al. investigated a polyethylene glycol (PEG) hydrogel modified with electrospun polycaprolactone (ePCL) [53]. This combines the biocompatibility of PEG and the strength and anisotropy from ePCL fibers, resulting in a structure that promoted a more typical anisotropic behavior in seeded valvular interstitial cells. Others are investigating woven nanofibers encapsulated within a hydrogel, to mimic the structure of cardiac tissue and control cellular alignment and elongation [54,55].

### 5.2. Bone Applications

Bone is a natural composite made up of organics and inorganics, predominately collagen (30%) and hydroxyapatite (70%) respectively [56]. Many calcium based ceramics such as hydroxyapatite, tricalcium phosphate, biphasic calcium phosphate, and calcium sulfate have been used in bone regeneration [57]. These materials facilitate new bone growth but are limited because their delivery can often require invasive procedures and they do not resemble tissues until they are combined with an underlying hydrogel matrix. Most composite hydrogels for bone tissue regeneration contain apatite or apatite-like minerals mentioned above, or have also been synthesized with the use of bioactive glasses or carbon nanotubes (CNTs). In all of these cases, a significant need is to establish composites with improved mechanical properties for the physical environment seen in bone. 

Dhivya et al. studied one such composite hydrogel, an injectable thermosensitive gel, incorporating nano hydroxyapatite (nHAP) particles into zinc-doped chitosan and beta-glycerophosphate (β-GP). Chitosan is widely used in biomedical applications, zinc was added because of its antimicrobial properties, and β-GP allows a controlled hydrogel formation. They found that the addition of nHAP increased protein adsorption, controlled swelling, and that the gel was osteoconductive [58]. Increasing the nHap ratio leads to an increase in compressive strength, however, chitosan and nHap alone do not adequately match the compressive strength of bone [59].

Others have attempted to use carbon nanotubes to improve mechanical properties in composite hydrogels (chitosan, poly-propylene fumarate, alginate, poly-2-hydroxyethyl-methacrylate) for bone regeneration. CNTs are known for their high toughness and surface area in addition to their electrical conductivity. These former properties can allow for improved composite toughness, and in combination with applied bone specific growth factors, such as bone morphogenic proteins (BMPs), have been shown to create a more conducive environment to enhance bone regeneration while reducing the need for more invasive treatment modalities [60,61].

### 5.3. Tendon Applications

Another biomedical application for composite hydrogels is in the treatment of tendinopathy. Tendon injuries and disorders are inherently difficult to heal due to their limited vascularity and tendency to form adhesive scar formations. Currently, acute and chronic tendinopathy are treated with moderately effective reconstructive surgeries, anabolic steroids, and autologous growth factor injections [62]. Composite hydrogels provide a possible solution to aid in the acceleration of wound repair in tendons via tissue-engineering scaffolds that passively or actively promote regeneration. One recent example of incorporating composite hydrogels in tendinopathy is the use of microparticulate-reinforcing phases in polyethylene glycol (PEG)-fibrinogen adhesive-hydrogels [29,63]. Hydrogels that integrate PEG into their structures are suitable for tissue-regeneration matrices because they are inherently biocompatible, easily cross-linked, and have the potential for tailored mechanical properties and performance as controlled drug-delivery vehicles (i.e., growth factors and other active molecules) [64]. For example, the addition of silica or fibrin microparticles create composites with modified mechanical properties and tissue adhesivity. Implementing elements that can be controllably released such as nitric oxide (NO) or hydrogen peroxide (H_2_O_2_), which are naturally antimicrobial signaling molecules, can be vital in the soft tissue wound-healing processes.

Networked composite hydrogels that consist of both micro and nano particles also provide a means of accelerating tendon growth and regeneration [25]. To mimic the normal tenocyte microenvironment, Li et al. developed a biodegradable alginate matrix with clay particles incorporated in the porous matrix with the capacity for controlled drug release. The particulate phase forms a network composite through the assembly of clay nanoparticles to generate larger micro particles, which provide binding sites for biological drugs, and are homogeneously distributed within the alginate matrix. As the hydrogel degrades, the clay particles slowly and controllably release the drug into the surrounding pathological tissue to assist in wound healing. This networked multiscale hydrogel composite supplies the mechanical and physical properties required to promote stable wound healing as well as the ability to store and release pharmaceuticals at an injury site.

In another study, fiber-reinforced hydrogel composites were implemented to create scaffolds for tendon tissue engineering. To emulate tendon architecture and function, multilayered polycaprolactone and gelatin fiber-hydrogel composites have been synthesized via electrospinning and ultravoilet (UV) crosslinking [28]. Polycaprolactone (PCL) fibers provided the mechanical and structural characteristics of tendons while the gelatin fibers supplied a mimic of the distinctive microenvironment surrounding tenocytes. Another example of fiber-reinforced composite hydrogels consists of aligned, discontinuous fibrous secondary phase hydrogels composed of PEG dimethacrylate and acrylated-PEG-peptide monomers [8]. As previously discussed, PEG provides a suitable structure and possible drug-delivery mechanism for tendon engineering scaffolds. Peptide monomers present in the composite hydrogel promote the adhesion between tenocytes and the fibrous components. Combining these constituents, a composite hydrogel designed to mimic the tension-related micromechanics of healthy tendons was developed.

In a tendinopathic setting, composite hydrogels provide advantageous qualities that mimic cellular microenvironments to promote tendon growth and regeneration. For example, fiber-reinforced matrices increase the number of binding sites for cellular focal adhesions to assist in maintaining a stable mechanical framework during healing. In addition, constituents of composite hydrogels that influence the structural characteristics of the overall material can improve tenocyte proliferation by providing directionality and alignment during regeneration. Many tendon injuries are habitually associated with the formation of adhesive scar tissue that can inhibit ordinary function. Composite hydrogels have the potential to reduce these pathogenic formations by acting as controlled drug delivery vehicles for growth factors, inhibitors, and other pharmaceuticals. By inducing more physiological compliance when compared to monolithic materials, composite hydrogels promote more stable tendon wound healing in vivo as a result of distinct physical, mechanical, and biological properties.

### 5.4. Neural Applications

Another physiological system where the loss of the structure–function relationship can lead to catastrophic consequences is the nervous system. Composite hydrogels have the ability to be tailored to these highly sensitive areas to assist, for example, in the regeneration of new spinal and peripheral nerve connections. In this system, permanent cells are unable to successfully repair normal architecture and function due to the harsh environmental changes caused by the initial injury. The common goal of hydrogel research within the central nervous system is generating guided growth permissive matrices that match the unique nervous system environment [65].

Strokes are one of the leading causes of mortality and are the result of reduced blood flow through localized regions within the brain, triggering ischemic-induced alterations to the environment, and restricted function of the normal surrounding cells [66]. In an effort to minimize and reverse the effects of stroke, Tang et al. has attempted to use hydrogels to provide suitable conditions to promote cell growth within the ischemic area, since traditional surgical methods require critical care within a narrow window after the initial stroke event [66]. These studies are focused on creating a protein-incorporated, injectable hydrogel that not only performs as a suitable environment for nerve cell motility, but also as an ECM recognizable by astrocytes and oligodendrocytes. These cells are known to stimulate and regulate neurogenesis, migration, and the signaling cascade of the neural regeneration process [66]. 

Damage to neural tissue can easily occur outside of the brain and further down the central nervous system. A common effect of spinal cord injury is a decrease in control of motor skills throughout the body, possibly resulting paraplegia, or even quadriplegia. The scar tissue found within the impaired sites discourages axonal regeneration, hindering the rate of recovery [67]. Potential hydrogels to assist in axon regeneration are being constructed as composite fiber-reinforced conduits in an attempt to control the directionality of the axon regeneration within the spinal cord [67]. Nanofibers have also been used in an attempt to create ECM with preferred directionality to promote differentiation of stem cells though neural lineage pathways [68].

## 6. Conclusions

In conclusion, composite hydrogels with distinct mechanical, physical, chemical and biological properties provide favorable matrices for tissue engineering by both actively and passively promoting an environment conducive to tissue regeneration. By mimicking normal tissue structure–function relationships on multiple scales, composite hydrogels emulate natural cell microenvironments, promoting differentiation, growth, and proliferation. These environments introduce controlled static and dynamic mechanical cues that cells sense, interpret, and respond to, resulting in desirable cellular behavior such as the generation of secure adhesion sites to their surroundings and maintaining biomechanical homeostasis. Numerous composite hydrogels have been designed for implementation in varying biomedical contexts and have the potential to revolutionize tissue engineering as an applied therapy in a clinical setting.
